# Environmental determinants of Parkinson’s disease and attributable burden in Latin America

**DOI:** 10.21203/rs.3.rs-10221836/v1

**Published:** 2026-07-14

**Authors:** Daniel Teixeira-dos-Santos, Henry Mauricio Chaparro-Solano, Juan Felipe Duarte-Zambrano, Thiago Leal, Miguel Inca-Martinez, Emily Waldo, Alastair Noyce, Dario Sergio Adamec, Emilia Mabel Gatto, Bruno Lopes Santos-Lobato, Juliana dos Santos Duarte, Francisco Eduardo Costa Cardoso, Grace Helena Letro, Gonzalo Arboleda, Jorge Orozco, Beatriz Muñoz Ospina, Paola Medina-Cordero, Pedro Chana-Cuevas, Ximena Pizarro-Correa, Philippe Salles, David Aguillon, David Pineda, Susana Lissette Peña Martínez, Tatiana Ascencio, Maria Valentina Müller, Marcelo Merello, Florencia Nicole Wainberg, Pedro Braga-Neto, Deborah Rangel, Marcela Susana Tela, Evelin Álvarez-Herrera, Mayela Rodríguez-Violante, Amin Cervantes-Arriaga, Daniel Martinez-Ramirez, Artur Schuh, Carlos Rieder, Mario Cornejo-Olivas, Cintia Armas, Julia Rios-Pinto, Angel Medina-Colque, Koni Mejia-Rojas, Angel Viñuela, Ana Lucia Rosso, Marcelo Kauffman, Vitor Tumas, Gabriel Vilela, Vanderci Borges, Cesar Avila, Patricio Olguín, Alicia Colombo, Juan Cristobal Nuñez, Samuel Jorquera, Elias Fernandez-Toledo, Alejandra Lázaro-Figueroa, Juan Manuel Esquivias-Farías, Ingrid Estrada-Bellmann, Paula Reyes-Pérez, Alejandra Medina-Rivera, Sarael Alcauter, María Teresa Muñoz-Quezada, Ignacio Mata

**Affiliations:** Cleveland Clinic; Case Western Reserve University; Cleveland Clinic; Morehouse School of Medicine; Cleveland Clinic; Cleveland Clinic; Queen Mary University of London; Hospital Posadas; Hospital de Niños de la Santísima Trinidad; Hospital Ophir Loyola; Universidade Federal do Pará; Universidade Federal de Minas Gerais; Hospital PUC-Campinas; Universidad Nacional de Colombia; Fundación Valle del Lili; Icesi University; Fundación Valle del Lili; Universidad de Santiago de Chile; CETRAM; CETRAM; University of Antioquia; University of Antioquia; Universidad Dr. Andrés Bello; Universidad Dr. Andrés Bello; Hospital de Clínicas “José de San Martín”; Fundación para la Lucha contra las Enfermedades Neurológicas de la Infancia; Fundación para la Lucha contra las Enfermedades Neurológicas de la Infancia; Federal University of Ceará; Federal University of Ceará; Hospital General de Agudos Dr. Juan A. Fernandez; Central American Technological University; National Institute of Neurology and Neurosurgery; National Institute of Neurological Sciences; Hospital Angeles Valle Oriente; Federal University of Rio Grande do Sul; Federal University of Health Sciences of Porto Alegre; Scientific University of the South; National Institute of Neurological Sciences; Universidad Peruana Los Andes; Regional Health Directorate of Puno; EDMECON Continuing Medical Education; Manati Medical Center and Fundación Parkinson Puerto Rico; Hospital Universitário Clementino Fraga Filho; Hospital Ramos Mejía; Ribeirão Preto Medical School, University of São Paulo; Ribeirão Preto Medical School, University of São Paulo; Federal University of São Paulo; National Scientific and Technical Research Council and National University of Tucuman; University of Chile; University of Chile; Clínica Alemana Santiago and Hospital Clínico Universidad de Chile; University of Chile; University of Concepción; National Autonomous University of Mexico; National Autonomous University of Mexico; University Hospital; Universidad Nacional Autónoma de México; Universidad Nacional Autónoma de México; National Autonomous University of Mexico; University of Chile; Cleveland Clinic

## Abstract

We investigated environmental determinants of Parkinson’s disease (PD) and their attributable burden in a case-control study of 9,887 participants from nine Latin American countries. Logistic regression adjusted for age, sex, and country estimated associations, and population attributable fractions (PAFs) were calculated for modifiable risk factors. Weekly caffeine consumption (adjusted odds ratio [aOR] 0.77, *p* < 0.0001) and tobacco use for ≥ 5 years (aOR 0.83, *p* = 0.0009) were inversely associated with PD, whereas severe head trauma (aOR 1.35), occupational pesticide exposure (aOR 1.42), occupational metal exposure (aOR 1.29), rural living (aOR 1.60), and well water consumption (aOR 1.22) increased PD risk (all *p* < 0.05). Dose-response relationships were observed. The joint PAF was 13.7% (95% CI 9.8–17.3), with higher estimates in El Salvador, Brazil, Colombia, men, and individuals younger than 60 years. Sensitivity and lag analyses yielded consistent results.

## Introduction

Parkinson’s disease (PD) is among the fastest growing neurological disorders worldwide, and its burden is expected to rise substantially over the coming decades.^1,2^ Although population aging is considered the main driver of this projected increase,^2^ improved public awareness, more accurate diagnosis, and better disease recording may also contribute to the rising number of identified cases.^1^ However the relative contribution of these factors remains uncertain, and growing interest has focused on the potential role of environmental and other modifiable risk factors in shaping the global burden of PD.^3^ In Latin America, this issue is particularly relevant because community based data from several countries indicate that PD already represents an important and growing public health challenge in the region.^4,5^ Nevertheless, the factors shaping regional differences in PD burden, especially the possible contribution of environmental determinants, remain incompletely understood.^6^ This knowledge gap restricts the ability to identify prevention and public health priorities that are locally relevant across countries and subgroups.

Many studies have linked PD to environmental and behavioural exposures, supporting the view that potentially modifiable factors may shape part of PD risk.^7^ However, most evidence comes from higher income settings, whereas Latin American populations remain underrepresented in PD research.^8^ This gap is relevant because the region has distinct agricultural, occupational, rural, and environmental contexts that may influence exposure patterns.^9,10^ These factors are part of the regional exposome, defined as the totality of environmental exposures experienced across the life course.^3^ In addition, the highly admixed genetic background of Latin American populations may contribute to regional differences in PD susceptibility and gene environment interactions.^11^ Generating region specific evidence is therefore important to identify locally relevant risk factors and prevention priorities.

Few studies have evaluated multiple environmental exposures in Latin American populations with PD, and their potential attributable burden across countries and demographic subgroups remains unknown. Population attributable fraction (PAF) can complement association estimates by quantifying the potential contribution of risk exposures under explicit modeling assumptions. We therefore evaluated associations between multiple participant-reported environmental exposures and PD in a large multicenter Latin American case-control cohort and estimated model-based PAFs for selected sustained risk exposures overall and across countries, sex, and age groups.

## Results

### Demographic and clinical characterization

The analytic sample included 9887 participants from nine Latin American countries, comprising 5729 participants with PD and 4158 controls. Baseline comparisons showed that participants with PD were older than controls, mean 64.1 vs 59.9 years, were more often male, 56.8% vs 31.3%, had fewer years of education, 11.6 vs 13.3, were more likely to report a positive family history of PD, 24.9% vs 13.2%, and had lower MoCA scores, 21.2 vs 23.9, all p < 0.001 (**Table 1**). Among PD cases, mean disease duration was 7.3 years, SD 6.4, and the median age at diagnosis was 57 years, IQR 18. Additional information on presenting symptoms, supportive PD features, and non-motor symptoms is provided in **Supplementary Table 1**.

**Figure 1:**
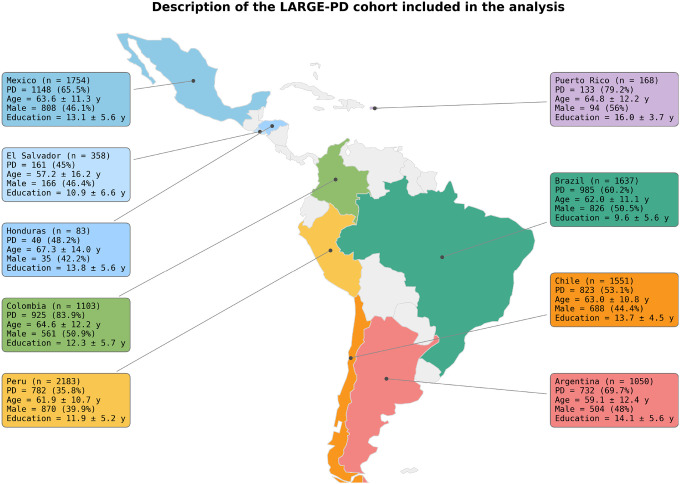
Adjusted associations between environmental exposures and PD. Forest plot showing adjusted odds ratios (aORs) and 95% CIs for environmental exposures associated with PD. Squares represent adjusted ORs, and horizontal lines represent 95% CIs. Green markers indicate inverse associations and red markers indicate positive associations; the vertical dotted line marks the null value (OR = 1). Models were adjusted for age, sex, and country. Only exposures with statistically significant adjusted associations are displayed. Exposures not reaching statistical significance in the adjusted analyses were tea consumption per 10 years, tea consumption ≥ 5 years, soda consumption per 10 years, soda consumption ≥ 5 years, alcohol consumption per 10 years, alcohol consumption ≥ 5 years, pesticide exposure outside of work per 10 years, pesticide exposure outside of work ≥ 5 years, years living close to a farm per 10 years, and living next to a farm ≥ 5 years.

Cross-country comparisons showed differences in demographic and clinical characteristics in the overall sample. The proportion of enrolled participants who were PD cases ranged from 35.8% in Peru to 83.9% in Colombia. Mean age ranged from 57.2 years in El Salvador to 67.3 years in Honduras, and mean years of education ranged from 9.6 years in Brazil to 16.0 years in Puerto Rico, all p < 0.0001 (Fig. 1, **Supplementary Table 2**). Among participants with PD, mean disease duration ranged from 5.0 years in Honduras to 10.1 years in Brazil, and positive family history of PD ranged from 14.3% in El Salvador to 34.4% in Chile, both p < 0.0001. Years of education were highest in Puerto Rico and Argentina and lowest in El Salvador and Brazil, whereas MoCA scores were highest in Argentina and Chile and lowest in Honduras and Brazil, both p < 0.0001 (**Supplementary Table 3**).

### Environmental associations with PD

**Figure 2:**
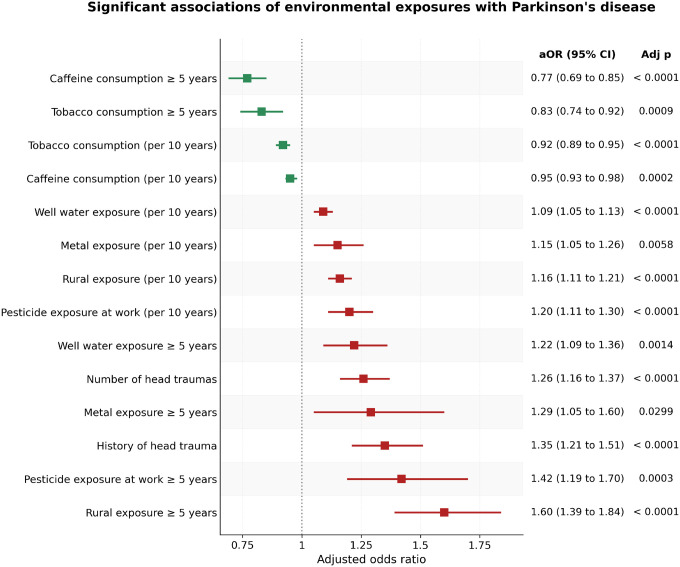
Description of the LARGE-PD cohort included in the analysis

After adjustment for age, sex, and country and correction for multiple comparisons, caffeine and tobacco consumption were inversely associated with PD, whereas occupational heavy metal exposure, head trauma, occupational pesticide exposure, rural living, and well water exposure were positively associated with PD (Fig. 2). Tea, caffeinated soda, alcohol, non-occupational pesticide exposure, and residential proximity to farms were not associated with PD in the primary analyses. Full estimates for all logistic regression models are presented in **Table 2**. Country-stratified logistic regression models and the cross-country exposure profile heatmap are shown in **Supplementary Figs. 1 to 10**.

Sensitivity analyses using lifetime ever exposure and cumulative dose measures are presented in **Supplementary Table 4**. Overall, the primary findings remained broadly consistent, with preservation of direction and statistical significance for caffeine, tobacco, occupational heavy metal exposure, occupational pesticide exposure, degree of urbanization of residence, and well water exposure. Dose-related patterns were also observed for caffeine, tobacco, pesticide exposure at work quantified as total days, tea intake, soda intake, degree of urbanization of residence, and well water exposure intensity. Among domains not associated with PD in the primary analyses, ever exposure to pesticides outside of work was inversely associated with PD after adjustment, whereas pesticide exposure outside of work quantified as total days and farm exposure remained non-significant. Lifetime alcohol use remained non-significant, whereas the cumulative alcohol exposure model was significant. **Supplementary Table 5** further shows that the main associations remained directionally consistent after restricting cases to those with more supportive PD features.

The null mixed model showed substantial clustering by study site, with an intraclass correlation coefficient of 0.334. After adjustment for country, the median intraclass correlation coefficient across mixed models decreased from 0.331 to 0.254, suggesting that part of the site-level heterogeneity was explained by country level differences. As shown in Supplementary Table 6, lag analyses based on age at PD diagnosis were feasible for alcohol, caffeine, farm exposure, head trauma, soda, tea, tobacco, urban exposure, and well water exposure, with high retention of exposed PD cases under both 1-year and 5-year lag definitions.

### PAF calculations

Five categorical exposures that remained statistically significant after confounder adjustment and multiple comparison control in the primary analyses were included in PAF analyses: (1) positive history of head trauma, at least 5 years of (2) rural living, (3) well water consumption, (4) occupational heavy metal exposure, and (5) occupational pesticide exposure. In the overall sample, the joint PAF was 13.7% (95% CI: 9.8% to 17.3%), with individual PAFs of 6.5% for head trauma, 6.0% for rural living, 4.2% for well water consumption, 2.5% for occupational pesticide exposure, and 1.1% for occupational heavy metal exposure.

**Figure 3:**
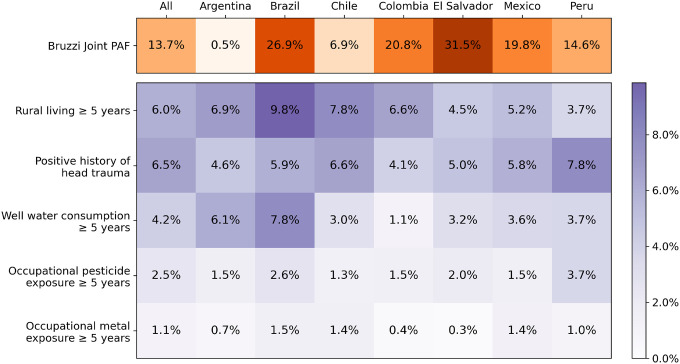
Country-specific PAFs for environmental exposures and PD Overall and country-specific joint PAF estimates and exposure-specific PAFs. Color intensity represents the magnitude of the estimated population attributable fraction, with darker shades indicating higher values. The orange scale represents the Bruzzi joint PAF across all included exposures, while the purple scale represents exposure specific PAF estimates. Values inside cells are percentages.

Country-specific PAF analyses showed substantial heterogeneity in attributable risk, with the lowest joint PAF observed in Argentina (0.5%) and the highest in El Salvador (31.5%) (Fig. 3). Higher estimates were also observed in Brazil and Colombia, at 26.9% and 20.8%, respectively, whereas Chile had a lower estimate of 6.9%. Across countries, rural living and head trauma were the most consistent major contributors. Brazil had the highest single exposure PAFs for rural living at 9.8% and well water consumption at 7.8%, with an additional contribution from head trauma of 5.9%. In contrast, Peru had the highest PAFs for head trauma and occupational pesticide exposure, at 7.8% and 3.7%, respectively. Occupational exposure to pesticides and heavy metals was generally small across countries.

**Figure 4:**
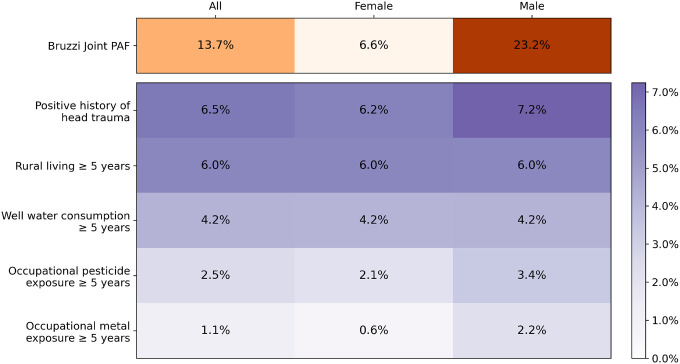
Sex specific PAFs for environmental exposures and PD Overall and sex specific joint PAF estimates and exposure-specific PAFs.

Sex-stratified PAF analyses showed substantial heterogeneity in attributable risk. Joint PAF was 6.6% in females and 23.2% in males (Fig. 4). Single exposure PAFs for head trauma, rural living, and well water consumption were broadly similar between sexes. By contrast, occupational contributions were larger in males, particularly for occupational pesticide exposure, 3.4% in males versus 2.1% in females, and occupational heavy metal exposure, 2.2% in males versus 0.6% of females.

**Figure 5:**
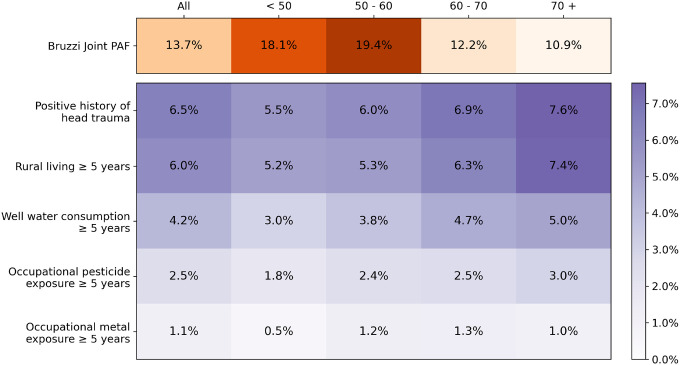
Age-specific PAFs for environmental exposures and PD Overall and age group-specific joint PAF estimates and exposure-specific PAFs

Age-stratified PAF analyses also showed heterogeneity in attributable risk. Joint PAF was higher in participants younger than 50 years (18.1%) and in those aged 50 to 60 years (19.4%) than in those aged 60 to 70 years (12.2%) or 70 years or older (10.9%; Fig. 5). In contrast, single exposure PAFs for head trauma, rural living, and well water consumption increased across older age groups. Among participants aged 70 years or older, PAFs reached 7.6% for head trauma, 7.4% for rural living, and 5.0% for well water consumption. PAFs for occupational pesticide and heavy metal exposure remained comparatively small across age groups.

**Supplementary Tables 7 to 9** provide additional details on the country, sex, and age group PAF calculations, including retained interaction terms. Sensitivity analyses using lifetime ever exposure for the five selected variables yielded patterns similar to the primary PAF estimates (**Supplementary Figs. 11 to 13**). In sensitivity analyses additionally adjusting for years of education (**Supplementary Figs. 14 to 16**) the overall joint PAF decreased from 13.7% to 10.8%, with the largest attenuations in El Salvador (31.5% to 19.0%), Brazil (26.9% to 23.9%), Mexico (19.8% to 13.6%), males (23.2% to 17.8%), participants younger than 50 years (18.1% to 9.1%), and those aged 50 to 60 years (19.4% to 16.1%). This reduction was mainly driven by attenuation of the rural living, well water, and occupational pesticide components, whereas head trauma remained similar. Further adjustment for PD family history and tobacco produced a comparable joint estimate of 10.5% (**Supplementary Figs. 17 to 19**).

## Discussion

In this large, multinational Latin American case-control study, we evaluated participant-reported environmental exposures in relation to PD and estimated the potential population impact of selected modifiable factors using PAF. After adjustment for age, sex, and country with multiple comparison control, caffeine and tobacco were inversely associated with PD risk, whereas occupational pesticide and heavy metal exposure, head trauma, rural living, and well water consumption were positively associated, most with dose-related patterns. The overall joint PAF was 13.7%, with the largest estimated contributions from head trauma, rural living, and well water consumption. PAFs varied across countries, sexes, and age groups, with the highest estimates in El Salvador, Brazil, and Colombia; approximately 3.5-fold higher estimates in males than females; and higher values in participants aged 60 years or younger. Findings were consistent across several sensitivity analyses, although additional adjustment for years of education yielded lower attributable risk estimates. To our knowledge, this is the first large multinational study in Latin America to jointly evaluate multiple environmental exposures associated with PD and to estimate their potential population impact using PAF.

With joint PAF estimates approaching or exceeding 20% in several countries, sex, and age strata, our findings suggest that sustained exposure to head trauma, rural living, well water consumption, occupational pesticide exposure, and occupational heavy metal exposure may contribute meaningfully to PD burden in Latin America. In a US deep south study evaluating overlapping exposure domains, the joint PAF among males was 30%, slightly higher than the 23.2% observed in our male stratum.^19^ Using a different set of exposures, a longitudinal UK Biobank cohort of 452,492 participants estimated that shifting individuals from less favorable to more favorable profiles across socioeconomic status, medical history, psychosocial factors, physical measures, and lifestyle could prevent up to 33.87% of PD cases^20^. These findings suggest that a nontrivial proportion of PD risk may be attributable to identifiable, potentially modifiable factors. This perspective is increasingly relevant because PD is projected to reach 25.2 million people worldwide by 2050, a 112% increase from 2021, with faster growth in lower sociodemographic settings such as Latin America.^2^ Identifying which environmental exposures most strongly shape PD risk across Latin American countries and subgroups may therefore help prioritize context-specific prevention strategies, guide occupational and environmental health policies, and plan health system responses to a growing regional burden.

Rural living deserves particular attention because it remained one of the largest individual PAFs in our study, was associated with multiple other environmental risk factors in pairwise analyses, and likely reflects a broader set of environmental contributors, including chronic exposure to agricultural toxicants, particularly pesticides.^7,21^ This interpretation is plausible in Latin America, where intensive agricultural activity, increasing pesticide use,^9^ and repeated detection of pesticides in groundwater have been documented across the region.^22^ At the same time, the absence of positive associations for farm proximity and non-occupational pesticide exposure in the primary analyses suggests that the rural living signal in our data is unlikely to be fully captured by a single self-reported agricultural exposure item and may instead reflect a broader environmental context. In Brazil, this pattern may be particularly relevant because rural living and well water consumption remained the strongest contributors to PD risk in our analysis, in a setting where groundwater quality remains incompletely characterized,^23^ and local studies have documented contamination of drinking water sources by pesticides and other hazardous contaminants.^24^ Similar examples of pesticide contamination in water sources have been reported in other Latin American countries, including Colombia^25^ and Chile.^26^

Head trauma also warrants particular attention as the largest individual contributor in the main PAF analysis and as another important regional contributor to PD risk, given the persistent burden of traffic-related, occupational injuries and interpersonal violence across Latin America,^27,28^ a region considered to have the highest incidence of traumatic brain injuries worldwide.^29^ This may be especially relevant in Peru, where the larger head trauma PAF could reflect the combination of a high road injury burden and population-based evidence of frequent lifetime head injury.^30,31^ Importantly, although higher head trauma incidence has been associated with lower socioeconomic status,^32^ adjustment for years of education, used here as an available proxy for socioeconomic position, resulted in the smallest PAF attenuation for head trauma. This suggests that the association was not fully explained by the socioeconomic dimension captured by educational attainment alone.

Beyond the largest overall PAFs, subgroup-specific patterns may identify additional prevention targets. In our study, this applied to occupational exposure to pesticides and heavy metals. Although both contributed less to overall attributable risk, they were more prominent among men, as was head trauma, suggesting that these exposures may cluster in occupations more commonly performed by men. This is relevant because the historical male predominance of PD has been increasingly reexamined, and part of this difference may reflect differential environmental and occupational exposure rather than only intrinsic biological susceptibility.^8^ In Latin America, this interpretation is plausible because men are more often directly involved in pesticide handling and protective practices are frequently inadequate, with studies from Argentina, Chile, Colombia, Brazil, Mexico, and Central America documenting low personal protective equipment use and unsafe handling conditions among agricultural workers.^33–38^ A similar pattern may apply to heavy metal exposure, given regional evidence of occupational lead and mercury exposure and literature showing that extractive work in Latin America remains strongly male-dominated.^39,40^ Taken together, these findings suggest that PD prevention in Latin America should also address subgroup-specific needs, including male-focused strategies in agricultural and extractive occupations, particularly safer handling protocols, biomonitoring, and stronger occupational health surveillance.^33,39^

It is noteworthy that the joint PAF was higher in participants younger than 50 years and in those aged 50 to 60 years than in older groups. This is particularly relevant because recent global data indicate that the burden of young onset PD has risen substantially,^41,42^ with incident cases more than tripling worldwide from 1990 to 2021. Although part of this increase may reflect improved recognition and access to diagnosis, it is unlikely to be explained entirely by ascertainment alone, because earlier onset forms of PD appear to be shaped by susceptibility beyond aging alone, including a higher prevalence of genetic contributors and a possible role for environmental exposures in accelerating clinical expression.^43,44^

Our findings therefore support the hypothesis that environmental exposures may be particularly relevant in PD occurring at younger ages, potentially contributing to earlier clinical expression in susceptible individuals. This interpretation is also consistent with the younger age profile of LARGE-PD, in which PD was diagnosed at a median age of 57 years, compared with large cohorts from Europe and the USA reporting mean age at diagnosis or onset in the early to mid-60s.^45,46^ From a public health perspective, this reinforces the importance of earlier environmental risk reduction, particularly in Latin America and other low and middle-income settings, where PD burden is rising, and the economic consequences of chronic neurological disability are especially difficult to absorb.^41,47^ In this context, previous projection-based studies suggesting that reducing harmful pesticides could prevent a meaningful number of future cases also supports the view that early prevention may be cost-effective.^48^

In our study, low joint PAF estimates were observed in two country stratified analyses, Argentina and Chile, and in the sex stratified analysis among females. Several explanations may account for these findings. First, several measured exposures were correlated with one another, as previously reported,^49^ so part of the lower joint PAF may reflect overlap among environmental domains. Joint modeling accounts for these interrelationships and may therefore yield a smaller attributable fraction than some individual PAF estimates. Second, these groups may have had a different susceptibility profile. Argentina and Chile had the highest educational attainment in both cases and controls. Because education attenuated PAF in our models, this pattern may reflect a more favorable socioeconomic and life course profile with less burden attributable to the environmental domains we assessed. These countries also had the highest frequency of positive family history, suggesting that inherited susceptibility may account for a larger proportion of PD risk in these groups. This interpretation is consistent with LARGE-PD evidence that genetic architecture varies across Latin American populations according to ancestry, including differences in the frequency of *LRRK2* variants associated with European ancestry.^11^ Finally, PD risk is shaped by a broader exposome than the one we assessed, and the lower joint PAF observed in these groups may partly reflect exposure profiles not captured by our measures, including physical activity, air pollution, diet, and, in females, reproductive factors related to lifetime estrogen exposure.^50–53^

Overall, most primary associations between environmental exposures and PD risk were directionally consistent with prior literature, including inverse associations for coffee consumption and tobacco use, and positive associations for occupational pesticide exposure and head trauma. The association with occupational heavy metal exposure was also partly compatible with previous evidence, although the literature remains less consistent.^54,55^ By contrast, rural living and especially well water consumption remain more heterogeneous domains, as broad reviews have described these associations as inconsistent, and a recent meta-analysis found no overall association for well water consumption.^56,57^ In our study, however, both rural living and well water consumption were positively associated with PD risk and remained positive across multiple exposure definitions, including dose-related metrics. One possible explanation is that well water may represent heterogeneous exposures across settings, including agricultural pesticide contamination or other local contaminants, which could differ between populations and contribute to variation across studies.

One possible explanation is that aggregated analyses may not fully capture Latin American exposure patterns, because environmental PD studies remain concentrated in Europe and the United States, and the well water literature is drawn mostly from Asia, Europe, and the United States.^57,58^ Consequently, contaminant mixtures and exposure circumstances more relevant to Latin America may be underrepresented in pooled estimates and in studies not based in the region. In addition, questionnaire-based variables may capture these exposures with limited precision, either because relevant thresholds are not adequately represented or because self-reported measures incompletely reflect the intensity, frequency, and context of exposure. Analyzing these variables too homogeneously across Latin American settings with distinct environmental, agricultural, and household exposure patterns may dilute associations that are more detectable in specific local contexts. This heterogeneity may also operate within Latin America, as suggested by household pesticide exposure, for which we did not observe a positive association in the overall cohort, in contrast to a prior Brazilian study.^18^ In parallel, dementia studies from Brazil and from seven Latin American countries have shown marked heterogeneity in attributable risk across settings, suggesting more broadly that neurodegenerative risk structures derived mainly from high income settings outside Latin America may not fully represent populations in the region.^59,60^

A major strength of this study is its large multinational Latin American design, which enabled the joint evaluation of multiple environmental exposures in relation to PD across countries, sexes, and age groups, with several sensitivity analyses showing broadly consistent results and supporting the robustness of the main findings. The use of PAF also adds a useful population perspective that may help identify relevant prevention targets in the region.

However, several limitations should be considered. First, exposure data were self-reported, making the findings vulnerable to recall bias and exposure misclassification. For exposures linked to place of residence or work, future studies using geolocation based exposure assessment, biomarker based approaches, direct toxicological analyses of biological specimens or environmental samples may reduce recall related bias, and strengthen temporal inference.^61,62^ Second, although data collection was harmonized across sites, differences in recall, interpretation of questionnaire items, and local meanings of environmental and occupational exposures may have contributed to between country heterogeneity, an observation supported by our exploratory mixed effects analyses suggesting substantial site level heterogeneity, partly explained by country level differences. Third, comparability with previous studies is limited by the broader lack of standardized environmental assessment in PD research, where exposures are often defined and analyzed differently across studies. We sought to mitigate this by evaluating multiple exposure definitions, including lifetime exposure, exposure for at least 5 years, years of exposure, and dose-related metrics, and by performing sensitivity analyses. In this context, ongoing Global Parkinson’s Genetics Program (GP2) efforts to strengthen epidemiological data collection and incorporate environmental risk factors in a more harmonized manner are especially relevant, and the availability of these data through GP2 may allow future replication using alternative definitions and harmonized workflows.^3,63^

Fourth, the environmental domains assessed do not capture the full exposome and genetic data were not incorporated. This is particularly relevant in PD, in which both environmental exposures and genetic susceptibility contribute to risk, and understanding their interaction is essential for a more complete etiological model. Because genetic risk likely varies across Latin American populations, integrating environmental and ancestry-informed genetic data represents an important next step to identify susceptible subgroups.^3,64^ Fifth, PAF should be interpreted cautiously because it is a model based measure that depends on causal assumptions. The case-control design limits temporal inference, and reverse causation cannot be fully excluded, particularly for behavioral exposures, despite the persistence of associations in the lag sensitivity analysis. Exposure specific PAFs are also approximate because Levin calculations used adjusted odds ratios as approximations of relative risks. Finally, because the consortium sample is not population representative, PAF estimates should not be interpreted as directly observed preventable fractions for Latin America, and may have been influenced by differences in control recruitment, study structure, referral pathways, and diagnostic access across countries.

In conclusion, our findings might suggest that a meaningful fraction of PD in Latin America may be linked to identifiable environmental exposures, particularly head trauma, rural living, well water consumption, and occupational pesticide and heavy metal exposure, with varying contributions across countries and subgroups. In particular, the higher joint PAF observed in younger participants and males suggests that prevention strategies should prioritize subgroups in which the attributable burden appears greatest. These results support placing environmental risk closer to the center of the regional PD agenda, not only as a research priority but also as a potentially actionable public health target. In practical terms, these findings support earlier attention to environmental and occupational exposures that may accumulate across the life course and contribute to PD risk in susceptible individuals. Strengthening surveillance of drinking water quality, agricultural and industrial exposures, and occupational safety may help guide prevention-oriented research and inform future public health strategies in Latin America.

## Methods

### Study design and setting

The Latin American Research Consortium on the Genetics of PD (LARGE PD) is a multicenter observational case-control study that has enrolled participants with PD and controls from 13 Latin American countries, across 52 sites, since 2005, using a standardized protocol and questionnaires.

LARGE-PD uses a standardized protocol and questionnaires across sites. The protocol includes (1) determination of eligibility and PD status based on the UK Queen Square Brain Bank (QSBB) criteria,^12^ (2) collection of harmonized demographic, clinical, and family history data from cases and controls, (3) a participant-reported environmental questionnaire capturing lifetime exposures, and (4) blood and/or saliva collection for genetic analyses. Participants were continuously recruited locally at each site through outpatient clinics and local outreach. Cases and controls were required to be aged 18 years or older. Patients were included if they fulfilled the diagnostic criteria for PD and had no exclusion criteria suggestive of alternative diagnoses, such as atypical or secondary parkinsonism. Controls were included if they were unrelated to enrolled cases and had no evidence of major neurological disease.

This study was coordinated through the LARGE-PD consortium, and all participating centers obtained local ethics committee or institutional review board approval, as applicable. All participants provided written informed consent, and analyses used deidentified data in accordance with the Declaration of Helsinki. This study was approved by the Cleveland Clinic Foundation Institutional Review Board (IRB No. 25–081).

### Variables

For all participants, we collected demographic and background information, including age, sex, years of education, self-reported ethnicity, family history of PD, and Montreal Cognitive Assessment (MoCA) scores.^13^ For participants with PD, clinical information included disease duration in years, age at diagnosis, Hoehn and Yahr stage,^14^ initial motor symptom, participant-reported lifetime non-motor symptoms lasting at least 1 month, and the presence of eight supportive clinical features from the QSBB criteria for PD diagnosis.^12^

Environmental exposures were assessed using a participant-reported lifetime questionnaire that captured exposures occurring at any point during life, with timing recorded when available through age at initiation, age at cessation, duration, or life stage specific histories. Participants reported tobacco, alcohol, and caffeinated beverage use, including age at initiation, age at cessation when applicable, and duration within prespecified intake categories. Severe head trauma was defined as a lifetime head injury associated with loss of consciousness or amnesia. Occupational exposures included self-reported metal exposure and work involving pesticide mixing or application, with information on duration and frequency. We also assessed non-occupational pesticide exposure and residential proximity to farms, defined as living within 400 metres of a farm. Residential history and usual drinking water sources were reported across prespecified life stages and used to derive rural living and well water exposure. Across domains, exposures were parameterized as duration per 10 years, duration of at least 5 years, lifetime ever exposure, and, when available, intensity based measures. Detailed information on all variables collected in this study is provided in the Supplementary Data.

In the analytic dataset, cases and controls were required to have complete demographic information, including age and sex, and non-missing data for all prespecified environmental exposure variables. To reduce the risk of unstable country-specific estimates due to sparse data, PAF analyses were additionally restricted to countries contributing at least 100 cases and 100 controls.

### Statistical analyses and population attributable fraction calculations

Group comparisons used chi-square tests for categorical variables and t tests or Mann-Whitney U tests for continuous variables, as appropriate. Associations between each environmental exposure and PD status were estimated using logistic regression. For each exposure domain, we evaluated two complementary exposure metrics in univariable and multivariable models: a binary parameterization and a continuous duration measure. Multivariable models were adjusted for age, sex, and country to estimate pooled Latin American associations while accounting for between country differences in recruitment structure, exposure prevalence, and case-control composition. Country was entered as a single categorical covariate, with one level for each country, and was treated as a fixed effect in the multivariable models. Exposures were modelled using two primary parameterizations: exposure duration of at least 5 years and duration per 10-year increment, with the latter representing the change in odds associated with each additional 10 years of reported exposure. We prioritized the ≥ 5-year definition over ever exposure because it better captured sustained exposure and reduced misclassification from brief or sporadic contact.

Multiple comparison correction across the environmental exposure set was performed using the Benjamini-Hochberg false discovery rate. Statistical significance was defined as p ≤ 0.05. Prespecified sensitivity analyses restricted cases to participants with at least five supportive QSBB features,^12^ and evaluated lag definitions by quantifying the proportion of exposed cases whose exposure preceded PD diagnosis by at least 1 year and 5 years. Between-site heterogeneity was assessed using mixed effects models with study site as a random intercept, comparing models before and after adjustment for country as a categorical fixed effect.

PAF was estimated for binary indicators of sustained risk exposures lasting at least 5 years that remained significant in the final multivariable models, as model-based estimates of potential attributable burden. Protective exposures were excluded from the main PAF framework because the analysis focused on potentially preventable harmful exposures. Exposure specific PAFs were calculated using Levin’s formula, with adjusted odds ratios used as approximations of relative risks and exposure prevalence among controls used as an estimate of source population prevalence.^15,16^ Joint PAF across the selected risk exposures was estimated using the Bruzzi method for case-control data.^17^ PAFs were computed overall and by country, sex, and age group in the restricted subset of countries contributing at least 100 cases and 100 controls. Sensitivity analyses repeated PAF estimation using lifetime ever exposure definitions and alternative adjustment sets, first adding years of education and then adding family history of PD and tobacco, as suggested in prior work.^18^ Analyses were conducted in Python version 3.11.2 using pandas, scipy, and matplotlib.

## Supplementary Material

Tables 1 and 2 are available in the Supplementary Files section.

Supplementary Files

This is a list of supplementary files associated with this preprint. Click to download.

- Table1.xlsx
- Table2.xlsx
- SupplementaryDataSupplementaryTables19.xlsx
- SupplementaryDataSupplementaryFigures119.docx
- ListofLARGEPDConsortiaMembersMainAuthorsandConsortiumAuthors.xlsx

## Data Availability

Deidentified participant data and the corresponding data dictionary will be made available with publication through the Global Parkinson’s Genetics Program (GP2), and the AMP PD Knowledge Platform, available at https://amp-pdrd.org/. GP2 data are accessible through the AMP PD platform, Terra, and Verily Workbench after completion of the required data access request and data use agreement, as applicable.

## Code availability

The Python code used for data harmonization, statistical analyses, population attributable fraction calculations, and figure generation will be made available to editors and reviewers upon request and will be deposited in a public repository upon publication, subject to consortium data governance requirements.
